# Molecular Design of Interfaces of Model Food Nanoemulsions: A Combined Experimental and Theoretical Approach

**DOI:** 10.3390/antiox12020484

**Published:** 2023-02-14

**Authors:** Tamara Martínez-Senra, Sonia Losada-Barreiro, Jose M. Hermida-Ramón, Ana M. Graña, Carlos Bravo-Díaz

**Affiliations:** Departamento Química-Física, Facultad de Química, Universidade de Vigo, 36310 Vigo, Spain

**Keywords:** gallic acid, nanoemulsions, molecular dynamics simulation, pseudophase kinetic model, distribution, lipid oxidation

## Abstract

The composition and structure of the interfacial region of emulsions frequently determine its functionality and practical applications. In this work, we have integrated theory and experiments to enable a detailed description of the location and orientation of antioxidants in the interfacial region of olive-oil-in-water nanoemulsions (O/W) loaded with the model gallic acid (GA) antioxidant. For the purpose, we determined the distribution of GA in the intact emulsions by employing the well-developed pseudophase kinetic model, as well as their oxidative stability. We also determined, by employing an in silico design, the radial distribution functions of GA to gain insights on its insertion depth and on its orientation in the interfacial region. Both theoretical and experimental methods provide comparable and complementary results, indicating that most GA is located in the interfacial region (~81.2%) with a small fraction in the aqueous (~18.82%). Thus, GA is an effective antioxidant to inhibit lipid oxidation in emulsions not only because of the energy required for its reaction with peroxyl radical is much lower than that between the peroxyl radical and the unsaturated lipid but also because its effective concentration in the interfacial region is much higher than the stoichiometric concentration. The results demonstrate that the hybrid approach of experiments and simulations constitutes a complementary and useful pathway to design new, tailored, functionalized emulsions to minimize lipid oxidation.

## 1. Introduction

There is an increasing tendency to use natural ingredients in product formulation, but this “green” evolution results in many opportunities and challenges for both industries and consumers. For instance, the food, pharmaceutical and cosmetic industries are continuously developing new functionalized products increasing the complexity of product formulation [1,2,3]. This is the case, for instance, of the preparation of food-grade oil-in-water (O/W) emulsions, which are prone to oxidation. Research during recent years has led to some progress in the control of their oxidative stability and current strategies to extend their shelf lives include the addition of natural antioxidants (AOs), which is frequently associated with the growing demand by consumers for healthier and more sustainable foods [4,5,6].

Food emulsions are thermodynamically unstable systems that can be stabilized kinetically for some time—minutes to years—by adding surface-active agents to prevent undesirable destabilization (e.g., flocculation, coalescence, etc.) phenomena [7,8]. The crucial region in these systems, where the inhibition reaction between antioxidants and peroxyl (ROO^●^) radicals mainly takes place, is the interfacial region of the emulsions [5,9,10]. Consequently, substantial efforts have been (and still are) made to characterize it and to understand how ROO^●^ and AOs are distributed and oriented in this highly anisotropic region.

In a series of papers [11,12,13,14], Losada-Barreiro et al. demonstrated the direct relationship between the efficiency of antioxidants in emulsions and their effective interfacial concentration, whose value depends on several factors, including the surfactant concentration, oil-to-water ratio, temperature, acidity, etc. [10,15,16,17]. Most probably, the most important is the amount of surfactant employed in the preparation of the emulsion because the addition of surfactants to stabilize the emulsion makes the antioxidants incorporate into the interfacial region of the emulsion, but at the same time, increases the interfacial volume. The increase in the percentage of antioxidant incorporated to the interfacial region does not compensate for the increase in the interfacial volume, resulting in a net decrease in the interfacial concentration of the antioxidants. Other factors that also modify the interfacial concentration of antioxidants include changes in acidity, temperature and the hydrophobicity of both the antioxidant and the surfactant [18,19,20].

Parmar et al. [21] were able to determine the location and orientation of several phenolic antioxidants in pluronic 104 micelles by employing two-dimensional nuclear Overhauser enhancement spectroscopy (2D-NOESY), a non-invasive method that provides important information on the location and the resulting chemical microenvironment of the antioxidants in micellar solutions. More recently, on the basis of EPR (electron paramagnetic resonance) experiments, Aliaga et al. [10,22] proposed that the relative orientation of the antioxidants and of the radicals in the interfacial region is associated with the structural characteristics of these molecules and with their amphiphobicity, playing an important role in determining the antioxidant efficiency. The authors demonstrated that the relative orientation of antioxidants and of the model TEMPO (4-alkanoyloxy-1,1,6,6-tetramethylpyperidineoxyl) radicals of different hydrophobicities controls the proximity of the reactive moieties. They concluded that in heterogeneous interfaces, a competition is established between the reactive groups for the hydrophobic core of a micelle or emulsion droplet, leading to distinct orientations and degrees of insertion into the lipophilic core [10,16,22,23].

Certainly, a proper characterization of the components present in the interfacial region is the basis for a successful formulation of new bio-based emulsions. Emulsion properties can be quantified using in silico approaches, but in vitro measurements are also necessary to confirm computational results. At the molecular scale, molecular simulation techniques (molecular dynamics (MD), Monte Carlo, etc.) constitute powerful tools to obtain insights into the dynamic properties, at the molecular level, of the interaction energies between molecules on the basis of statistical mechanics. Specifically, MD is capable of simulating colloidal systems in which atoms and molecules interact during a defined time period according to their physicochemical properties, determining, for example, the physical emulsion stability via free-energy calculations [23,24,25].

Molecular dynamics simulation (MDS) techniques may also provide new insights about the puzzling antioxidant–radicals interactions and their relative orientations. For instance, Aliaga et al. [10,23] employed MDS to obtain insights into the relative orientation of a series of 4-substituted TEMPO derivatives in Triton-XR 100 micelles, disclosing that the various 4-substituted TEMPO radicals are inserted and oriented in different positions and angles depending on their hydrophobicity. The authors reported that the variation of the distance between the center of the micelle and the -NO^●^ group of the TEMPO derivatives with the length of the alky chain of the TEMPO derivative follows a cut-off-like pattern. That is, the distance decreases upon increasing the length of the alkyl chain until it reaches the octyl derivative, after which a further increase in the hydrophobicity leads to an increase in the distance [10,22].

Here, we report the first results of a long-term collaborative project aimed to increase our knowledge about the position and orientation of antioxidants in O/W nanoemulsions by bringing together experimental and theoretical results. In this paper, we describe the MDS technique employed and compare the results obtained for the gallic acid (GA) with those obtained experimentally. We also investigated the fate of the reaction between GA and peroxyl radicals theoretically, and determined the energy of the transition state in order to calculate the energy barrier, which, according to the theory of absolute rates, is related to the rate constant of the inhibition reaction [26,27]. Results also allowed us to analyze the relationships between the location and/or orientation of the antioxidant and their efficiency in inhibiting lipid peroxidation.

Our long-term aim focuses, as in previous works, on understanding the complex behavior of antioxidants in emulsified systems to develop a scientific basis that allows a better comprehension of their behavior in emulsions, in order to predict the antioxidant, or combinations of antioxidants, with the best efficiency, and to design better strategies to control the undesirable lipid oxidation reactions.

Nowadays, quantum-mechanics-based and molecular simulation technologies have an important role in chemical research, and combined theoretical–experimental approaches are currently employed to address specific chemical problems, for instance, MD simulations are used routinely, to give atomistic-level insights into the dynamical and structural properties of compounds in multiphasic systems, information that cannot be otherwise obtained because of experimental difficulties [24,28,29,30]. Particularly, they may provide valuable quantitative information on lipid oxidation reactions and their inhibition in the interfacial region of emulsions. Their role in contemporary chemistry is certainly affected by the accuracy of the results obtained, making combinations of wet-lab experiments with MD simulations provide, very frequently, synergistic results to enhance our knowledge on the control of lipid oxidation reactions by antioxidants or other specific chemical problems [24,31,32,33,34].

We are aware that, in spite of the enormous progress of computational methods in recent years, they cannot still describe the enormous complexity of food or biological systems. Olive oil contains a number of fatty acids, but for the sake of computational simplicity, we simulated olive-oil-in-water emulsions in silico by employing exclusively oleic acid, which is the major fatty acid present (>72%, see Section 3.1 below). In spite of this simplification, which does not affect significantly the overall conclusions of the paper, our aim here is to provide basic information on the location and/or orientation of antioxidants in model food nanoemulsions, not to obtain insights into the nanoemulsions themselves because excellent books and reviews are available regarding their preparation, physical stability and applications. Certainly, we believe that the combination of both computational (in silico) and experimental (in vitro) methods will play a major role in successful bio-emulsion designs because we can take advantage of the predictive properties of their computational methods that are close to what is obtained experimentally. In addition, the combination of both paths (in silico and in vitro) will be less time-consuming than other trial-and-error methods and, thus, can be expected to be employed at all scales in industry and to be the major factor in successful bio-based emulsion design.

## 2. A Brief Summary of the Lipid Oxidation Reaction and Its Inhibition by Antioxidants in Emulsions

**Scheme 1:**
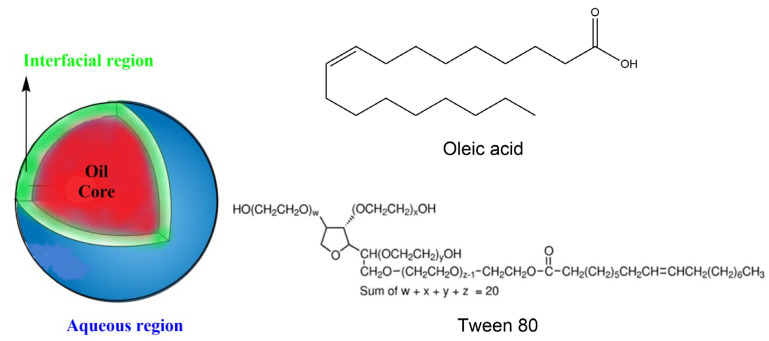
(**Left**) Typical spherical droplet of O/W emulsions showing the conceptual division of the different regions (aqueous, interfacial and oil). (**Right**) Chemical structures of oleic acid and Tween 80.

Lipids are present in a variety of natural and processed foods, including sauces, soft drinks, soups, etc., in the form of oil-in-water emulsions [35,36], which are thermodynamically unstable soft materials consisting of small droplets of oil (dispersed phase) in a continuous aqueous phase, Scheme 1. Emulsions can be stabilized kinetically by the addition of surfactants (polysorbates, sucrose esters, natural proteins, etc.) that accumulate in the three dimensional (2–50 nm thick) interfacial region.

The interfacial region is highly anisotropic, and it is composed of a melange of oil, surfactant and water [35], and many relevant biological, chemical and technological processes, including the oxidation of lipids, take place in this region. Because of its solvent properties, reactants may accumulate in the interfacial region, making their effective concentrations much higher than the stoichiometric ones because of the small interfacial volume, which is much smaller than those of the oil and aqueous regions.

**Scheme 2:**
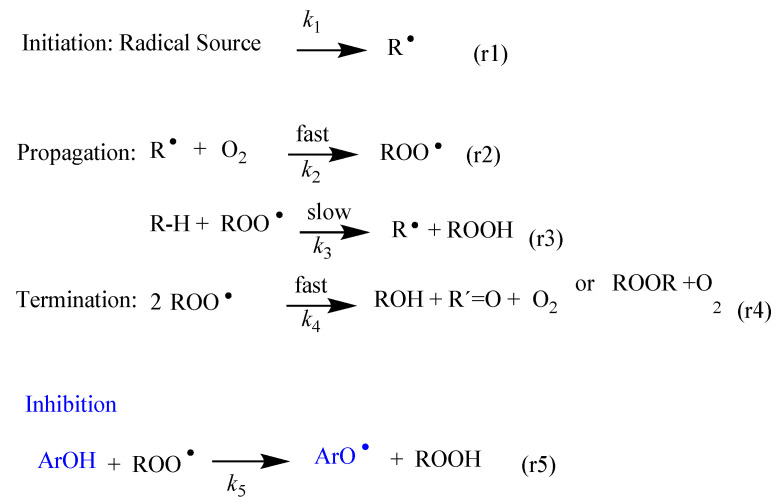
Basic steps (r1–r4) of the chain reaction in the oxidation of an unsaturated lipid (R-H). The inhibition reaction (r5) takes place upon the transfer of an H atom of a phenolic antioxidant (ArOH) to the chain carrying ROO^●^ radicals. R^●^, ROO^●^ and RO^●^ stand for alkyl, alkylperoxyl and alkoxyl radicals.

The lipid oxidation is a quite complex radical reaction [37,38,39,40,41] that can be, in short, described in terms of the initiation, propagation and termination steps indicated in Scheme 2. The reaction is not spontaneous and needs initiators such as light, heat, metals, enzymes, hydroxyl radicals, labile azo dyes or other reactive oxygen or nitrogen species that trigger the reaction by abstracting an H atom of unsaturated fatty acids (RH), leading to the formation of alkyl radicals, R^●^, Reaction 1 in Scheme 2. In the propagation step, the formed alkyl radicals R^●^ react rapidly with molecular oxygen to give peroxyl radicals (ROO^●^), whose structure depends on the nature of the fatty acids, Reaction 2 in Scheme 2. These new ROO^●^ radicals react with another RH molecule to form hydroperoxides (ROOH) and R^●^ radicals (Reaction 3 in Scheme 2). The new alkyl radical reacts again with molecular oxygen to form new peroxyl radicals and the process is repeated again. The reaction stops in the termination step when two free radicals combine to form stable products or when all the unsaturated fatty acids are consumed and no H-atoms can be abstracted (Reaction 4 in Scheme 2).

The production of hydroperoxides (ROOH) during the propagation step (Reaction 3 in Scheme 2) can be efficiently controlled by adding (poly)phenolic antioxidants, ArOH, which react with the peroxyl radicals ROO^●^ at rates higher than those of the propagation step, Reaction 5 in Scheme 2, opening a new reaction pathway.

The antioxidants donate and H atom to the peroxyl radical, leading to the regeneration of the parent lipid, and producing antioxidant radicals ArO^●^. The reactivity of ArO^●^ radicals is so low that they are not capable of abstracting H-atoms from the unsaturated lipids, and therefore, do not re-initiate the chain reaction. The literature reports indicate that the reaction between ArOH and ROO^●^ can take place through several (sometimes simultaneous) mechanisms, depending on the solvent and the chemical nature of the antioxidant; the most important and frequent is the hydrogen atom transfer (HAT) mechanism [39,42,43].

## 3. Materials and Methods

### 3.1. Materials

Commercial extra-virgin olive oil was purchased from a local shop and was stripped of its endogenous antioxidants, following a classic chromatographic method [13]. Briefly, olive oil, stripped of natural tocopherols and phenols, was prepared from commercial virgin olive oil by washing it with 0.5 M NaOH solution and passing it twice through an aluminium oxide column. The complete removal of tocopherols was confirmed by HPLC according to the IUPAC method 2.432. The content of tocopherols was measured to confirm the efficiency of the stripped process. Oleic acid is the primary component of the stripped olive oil (72.1%), as is shown in Table 1. The prepared stripped oil was transferred into a sealed amber bottle and kept at a low temperature to minimize its oxidation. Gallic acid and Tween 80 of analytical grade were purchased from Sigma-Aldrich (St. Louis, MO, USA). The aqueous phase was a buffered solution composed by citric acid and sodium citrate (Across Organics, Geel, Belgium). All other chemicals employed in this study were of analytical grade and supplied by Sigma-Aldrich or Across Organics.

### 3.2. Preparation of O/W Nanoemulsions

A coarse O/W emulsion containing 10% (*v*:*v*) stripped olive oil, different quantities of emulsifier and 90% (*v*:*v*) aqueous-citric-buffered solution was prepared by employing a high-speed rotor (Polytron PT 1600 E, 20,000 rpm for 1 min). Subsequently, the coarse emulsions were passed through a high-pressure microfluidizer (Microfluidics, LM20 Microfluidizer) working at 25,000 Psi (137.5 MPa), following the procedure descripted elsewhere [9,12,16,44].

### 3.3. Determination of ζ-Potentials and Average Droplet Sizes

**Figure 1:**
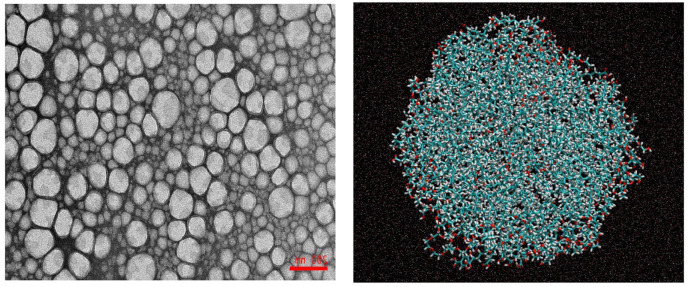
(**Left**) TEM microphotograph (15,000×) of stained (uranyl acetate) 1:9 (*v*:*v*) oil-in-water nanoemulsions, stabillized with Tween 80, Φ_I_ = 0.0378. (**Right**) Snapshot of an emulsion droplet obtained through computer simulation (water is omitted for clarity).

A Malvern Mastersizer MS 3000 (Malvern Instruments Ltd., Cambridge, UK) was employed to evaluate the droplet sizes and polydispersity indexes. ζ-potential values were estimated by employing a particle electrophoresis instrument (Zetasizer Nanoseries Nano-ZS, Malvern Instruments, Worcestershire, UK). Different emulsions were diluted 100 times with citric buffer before measurement. All measurements were performed in triplicate and reported values were given as average ± standard deviation. At Φ_I_ = 0.038, the average droplet size was 478 ± 58 nm. The polidispersity index (PDI) was 0.22, suggesting that nanoemulsions were essentially monodisperse. The ζ-potential of the nanoemulsions was −15 mV, according to the non-ionic nature of the surfactant Tween 80. Note that the destabilization process was not observed by the naked eye for more than 3 months. Figure 1 shows a TEM photograph of the nanoemulsions prepared in this work.

### 3.4. Oxidative Stability Experiments

The oxidative stability of the nanoemulsions (in the absence—control—and in the presence of GA) was evaluated by employing an accelerated oxidation assay (Schaal oven test) at T = 60 °C. The oxidative degree was evaluated by monitoring the formation of the primary oxidation products (conjugated dienes, CDs) with time according to published procedures [16]. The aliquots of the nanoemulsion sample were diluted to 10 mL with ethanol and the absorbance value was obtained at λ = 233 nm. %ΔCD was determined according to the AOCS Ti 1a-64 method [17].

The kinetic curves of the accumulation of conjugates dienes were obtained by plotting variations in the formation of CD with time. The oxidative stability of emulsion was quantified by determining the induction time (τ_IND_) in the absence and in the presence of GA [15]. τ_IND_ is the critical time at which the transition from the initiation to the propagation stage takes place, and was determined, as in previous works, by the intersection point of the two straight lines during the induction period and the propagation step of the reaction [15,16].

### 3.5. Determining the Antioxidant Distribution in Intact Nanoemulsions (In Vitro Method)

**Figure 2:**
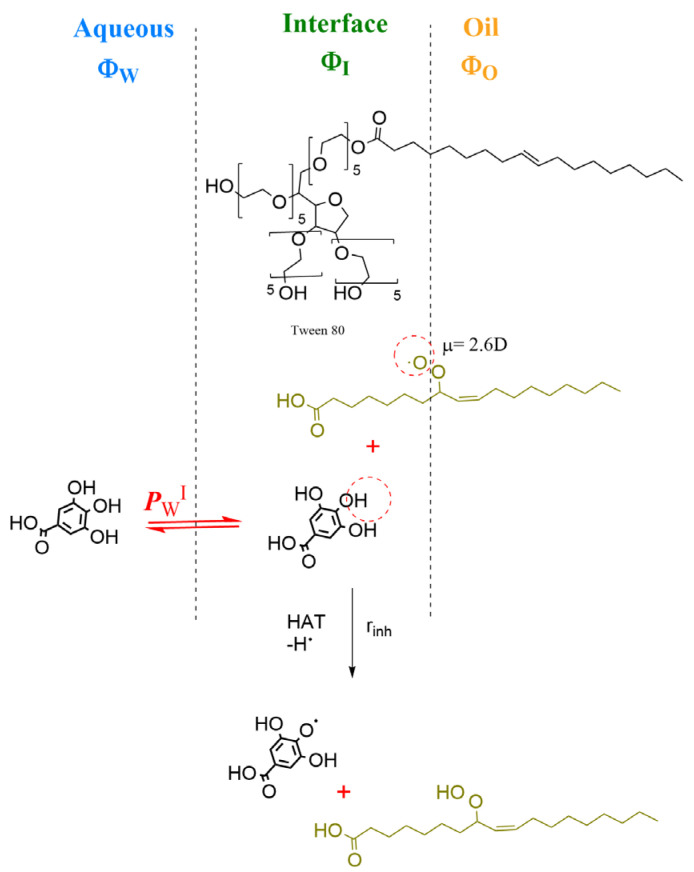
Two-dimensional representation of an emulsion droplet showing the aqueous, interfacial and oil regions and the chemical structures of the surfactant Tween 80, the antioxidant gallic acid GA and that of a peroxyl radical. The orientation of the reactants is illustrative. GA is oil-insoluble and only distributes itself between the aqueous and interfacial regions. The inhibition reaction (HAT mechanism) of GA with the peroxyl radicals mainly takes place in the interfacial region of the emulsion and through the HAT mechanism [42].

A kinetic method based on the pseudophase kinetic model was employed to determine the gallic acid distribution in the prepared nanoemulsions [45]. The method is grounded on the analyses of the variations of the observed rate constant, *k*_obs_, for the reaction between the chemical probe 4-hexadecylbenzenediazonium, 16ArN_2_^+^, and gallic acid with the emulsifier volume fractions Φ_I_ (=V_surf_/V_emulsion_) in intact nanoemulsions. Because of gallic acid is oil-insoluble, only the partition constant between the aqueous and interfacial regions, *P*_W_^I^, is needed to describe its distribution, Figure 2. *P*_W_^I^ is defined by Equation (1), and according to the theory of pseudophase kinetic model, Equation (2) can be derived from where *P*_W_^I^ can be obtained. Further details on the calculations and the derivation of Equation (2) can be found elsewhere [45]. Once *P*_W_^I^ was determined, the percentage of GA located at the interfacial region and its effective concentration (mol/L) were estimated by employing Equations (3) and (4).

(1) $$P_{W}^{I}=\frac{{(\mathrm{AO}}_{I})}{{(\mathrm{AO}}_{W})}$$

(2) $$k_{\mathrm{obs}}=\frac{\left[{\mathrm{AO}}_{T}\right]k_{I}P_{W}^{I}}{\Phi_{{}_{I}}P_{W}^{I}+\Phi_{W}}$$

(3) $${\%\mathrm{AO}}_{I}=\frac{{100\Phi }_{I}P_{W}^{I}}{\Phi_{W}{+\Phi }_{I}P_{W}^{I}}$$

(4) $${(\mathrm{AO}}_{I})=\frac{n_{I}}{V_{I}}=\frac{{[\mathrm{AO}}_{T}{](\%\mathrm{AO}}_{I})}{\Phi_{I}}$$

### 3.6. Computational Design: Molecular Dynamics Simulation of a Food-Grade Nanoemulsion

In this paper, and for the sake of computational simplicity, we simulated the olive oil nanoemulsion by employing exclusively oleic acid because it is the main component of the oil (Table 1). The initial configuration for simulating food-emulsions was obtained by employing Packmol software v20.2.3 [46]. The initial model nanoemulsion was built with 200 molecules of oleic acid and 20 molecules of Tween 80 (w = x = y = z = 5), all placed in a spherical configuration to simulate the oil core and interfacial region of the nanoemulsion (Scheme 1). Eight gallic acid molecules were then added randomly to the surface of the spherical droplet. The number of Tween 80 molecules employed is proportional to the expected number of molecules present in a 1:9 (*v*:*v*) O/W nanoemulsion.

The aqueous phase (solvating water) was simulated by employing the LEAP module of the AMBER (assisted model building and energy refinement) suit of programs [47,48] with a TIP3P water-truncated octahedron box surrounding the oily droplet so that the distance between any point of the oil droplet with any size of the octahedron box was not less than 23 Å. This distance was chosen to limit the aqueous volume and obtain reasonable computational calculation times. The total number of molecules employed in the simulations to model the oil-in-water nanoemulsion was: 33,804 molecules of water, 200 of oleic acid, 20 of Tween 80 and 8 of gallic acid, Figure 1. The relative number of oleic acid, Tween 80 and gallic acid simulates a 1:9 nanoemulsion containing a surfactant volume fraction Φ_I_ of 0.04 (Φ_I_ = V_surf_/V_emulsion_).

The generalized AMBER force field (gaff) parameters were used to describe the interactions between Tween 80, gallic acid and oleic acid molecules, and the TIP3P model [49] was used to describe the water molecules.

Before starting the MD simulations, the nanoemulsion geometry was minimized without restraints with the conjugate gradient method at 0 K using periodic boundary conditions in a NVT (constant number of particles, volume and temperature) ensemble to minimize the Brownian motion. Long-range electrostatic interactions were calculated in all MD simulations based on particle mesh Ewald’s method with a cut-off value of 12 Å. After the minimization of the geometry, a heating procedure was performed. Thus, a MD simulation was carried out for 12.5 ns, and the MD temperature was increased in a NPT (constant number of particles, pressure and temperature) ensemble from 0 K to 300 K to simulate the molecular motion of real emulsions. The time-step of this simulation was 1 fs and a Langevin thermostat collision frequency of 1.0 was used to control the temperature. The pressure was maintained at 1 atm by using the Berendsen barostat with a relaxation time of 2 ps. The SHAKE algorithm [50] was applied to constrain all bond interactions involving hydrogen. The Langevin thermostat, Berendsen barostat and SHAKE algorithm were also used in the following MD equilibration of the system. This simulation was performed for 40 ns in an NPT ensemble with a time-step of 2 fs. The same technical specifications of the equilibration were used in the production of the MD simulation that generated the conformations employed to perform the analysis. Thus, after the equilibration, the system was subjected to an MD simulation for 200 ns and the trajectory was stored every 2 ps. Stored configurations were analyzed using the cppTraj module [51] included in AmberTools. All MD simulations were performed using the AMBER package [48,52], and VMD [53] was used for the visualization of the structures and trajectories resulting from the MD simulations.

### 3.7. Probability Density Profiles of Nanoemulsion Components: Reference Points

Radial distribution functions, RDFs, describe how the density of surrounding particles (atoms, molecules, colloids, etc.) varies as a function of the distance (R) from a given point in a system. To describe the RDFs of the nanoemulsion components we have chosen, as reference point, the center of mass (COM) of the nanoemulsion droplet and the COMs of gallic acid, oleic acid and water molecules. To facilitate computational calculations, the Tween 80 molecule was divided in three portions so that the reference points for the molecule were: (1) The center of mass of the five-carbon ring; (2) The oxygen atoms of the hydroxyl moieties at the end of each ethylene oxide units; and (3) The hydrophobic oleate chain.

### 3.8. Computational Description of the Insertion Depth of Gallic Acid and Its Relative Orientation in the Interface of the Nanoemulsions

**Figure 3:**
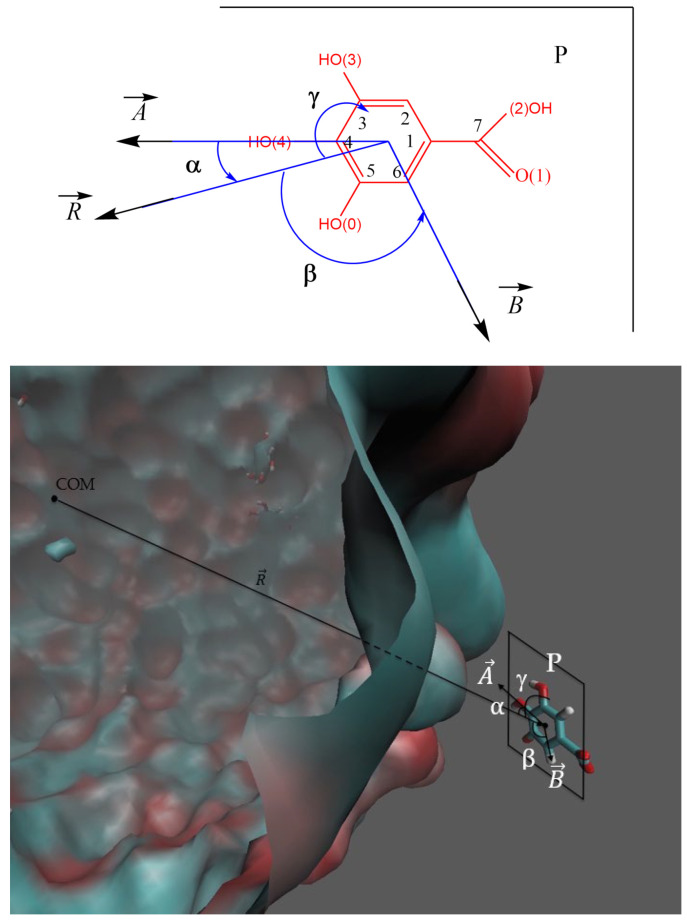
(**Upper**) Detail of the molecular structure of the gallic acid, numbering and vectors used in the simulation and its analysis. Angles and atom numeration established to analyze the orientation of the gallic acid with respect to the nanoemulsion droplet core. The α angle is that between the vector joining the COM of the nanoemulsion (vector $\vec{R}$) and the vector contained in the plane P, joining the center of mass of gallic acid and the C4 atom (vector $\vec{A}$). The β angle is that between vector $\vec{R}$ and vector $\vec{B}$ contained in the plane. The γ angle between vector $\vec{R}$ and the molecular plane P. (**Lower**) The computer-simulated picture of the modelled nanoemulsion (MD) and of the gallic acid molecule (both drawn keeping their relative sizes), and the main parameters employed to describe the location and orientation of GA in the nanoemulsion. The atoms of GA are represented according to the following color-coding: C (cyan), H (white), O (red).

The spatial distribution of GA is described in terms of the distance along the vector $\vec{R}$ from COM of GA to the COM of the nanoemulsion droplet, as illustrated in Figure 3. The relative orientation of GA in the nanoemulsion is described with the aid of the angles α, β and γ, Figure 3. The angle α stands for that between the vector $\vec{R}$ and the line defined by the atoms O(4)-C4-C1-C7, which is contained in plane P of the aromatic ring of GA (vector $\vec{A}$). The β angle defines that between vector $\vec{R}$ and vector $\vec{B}$ (joining the COM of GA and the C6 atom). The γ angle refers to that between vector $\vec{R}$ and the molecular plane, *P*. Thus, when the plane of the aromatic ring is parallel to the droplet surface then γ = 90 °C, while when perpendicular then γ = 0 °C.

### 3.9. Determining the Electronic Barrier for the Reaction between ROO^●^ and GA and between ROO^●^ and ROOH

All QM energy calculations were carried out by using a BLYP functional with Grimme dispersion correction and a def2-svp basis set. The ORCA package was used for the calculations. Constrained optimizations were performed by increasing the distance (reaction coordinate) between O4 and the H bonded to it (see Figure 3 for atom numbering) in 0.01 Å steps until the H atom bonds the terminal oxygen of the peroxyl radical (inhibition Reaction 5 in Scheme 2). For each distance, the remaining degrees of freedom were allowed to relax.

### 3.10. Some Technical Details for QM/MM Simulations

In order to choose the initial configurations for the QM/MM simulations, we considered a geometric threshold of 4 Å for the distance between the O4 of gallic acid and one of the carbons where the oxidation can occur in the oleic acid. This threshold was arbitrarily chosen to simulate real reaction conditions (the molecules need to form an encounter complex, and therefore, the involved atoms need to be close to each other) and to obtain reasonable computational times. All configurations obtained from the MD simulation that fulfilled the threshold distance were stored as if they were potential starting configurations for the QM/MM simulations. From all of them, we took four different configurations that encompass the whole MD simulation to perform the QM/MM simulations. Once the configurations were selected, the system that was to be simulated was defined according to the following protocol: the hydrogen bonded to the carbon of the oleic acid (the one used in our threshold) was substituted by the peroxy group; both the peroxyl radical and gallic acid were treated by a QM method in the QM/MM simulation; two spheres with radii of 12 and 20 Å (from the carbon atom where the peroxyl group is attached) were set so that any molecule inside the 12 Å radius sphere was included as part of the unrestrained region in the molecular mechanics description of the QM/MM calculations; molecules at distances between 12 Å and 20 Å were included in the fix region of the molecular mechanics description.

The QM part of the system was calculated by using a BLYP functional with a Grimme dispersion correction and a def2-svp basis set. The MM part of the system was calculated by using the same AMBER force field parameters that exist in the classical MD simulations and also considering the water molecules as rigid species. Systems were simulated for 10 ps or until the reaction occurred. On each simulation, a pre-equilibration was performed with a Berendsen thermostat during 50 fs, while a Nosé–Hoover chain (NHC) thermostat was employed during the remaining part of simulations [54,55]. QM/MM simulations were performed using the ORCA package.

## 4. Results and Discussion

### 4.1. Determining the Location and Distribution of GA in O/W Nanoemulsions

The location and distribution of GA in olive O/W nanoemulsions were determined both in vitro and in silico.

**Figure 4:**
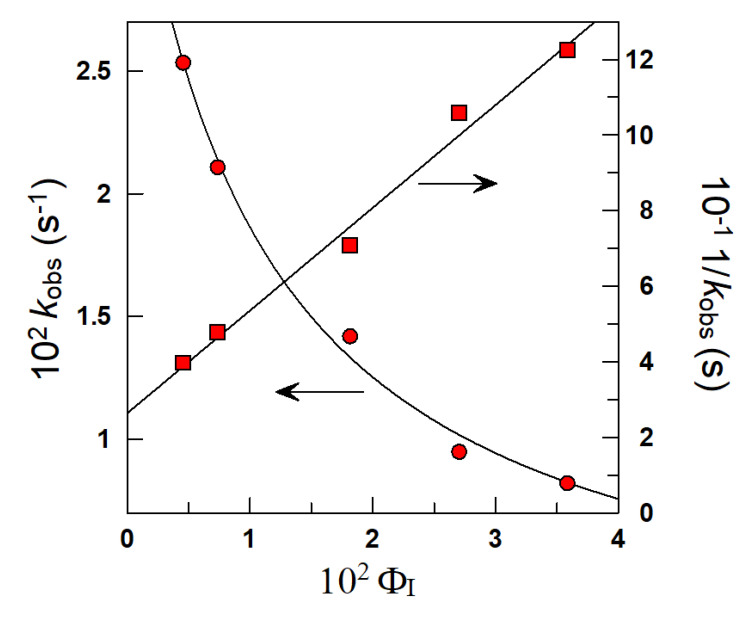
Variation of the observed rate constants with the emulsifier volume fraction (Φ_I_) for the reaction between chemical probe and GA in 1:9 (O/W) nanoemulsions. pH = 3.65 (citric-acid-citrate buffer 0.04 M), Tween 80, [16-ArN_2_^+^] = 1.4 × 10^−4^ M, [NED] = 0.02 M, [GA_T_] =3 × 10^−3^ M, T = 25 °C.

In vitro, the partition constant *P*_W_^I^ of GA was estimated in the intact nanoemulsions, as indicated in Section 3.5. Figure 4 shows the variation of *k*_obs_ and 1/*k*_obs_ with Φ_I_, and the experimental pairs of data (*k*_obs,_ Φ_I_) and (1/*k*_obs_, Φ_I_) were fitted to Equation (2). The calculated *P*_W_^I^ value was *P*_W_^I^ = 93 ± 11, a value that reflects the tendency of GA to be transferred spontaneously from the water to the interfacial region.

The distribution of GA between the aqueous and interfacial regions was determined by employing Equation (3). Table 2 shows the variations of the percentage of GA in the aqueous (%GA_W_) and interfacial (%GA_I_) regions of the nanoemulsion at the lowest and highest surfactant volume fractions employed in the preparation of the nanoemulsion. At the lowest Φ_I_ value employed, Φ_I_ = 0.005, GA was mostly located in the aqueous region (65.8%) and only 34.2% was located in the interfacial region. %GA_I_ increased upon increasing Φ_I_, so that %GA_I_ = 81.2 when Φ_I_ = 0.04.

Although GA is incorporated in the interfacial region upon increasing Φ_I_, its effective concentration decreases because of the increase in the interfacial volume, Equation (4). Results in Table 2 indicate the effective concentration of GA, (GA_I_), expressed in moles per liter of interfacial volume, which is 70- fold higher than the stoichiometric concentration [AO_T_] = 10^−4^ M (Φ_I_ = 0.005), and that this value decreases 3.5-fold when Φ_I_ = 0.04.

**Figure 5:**
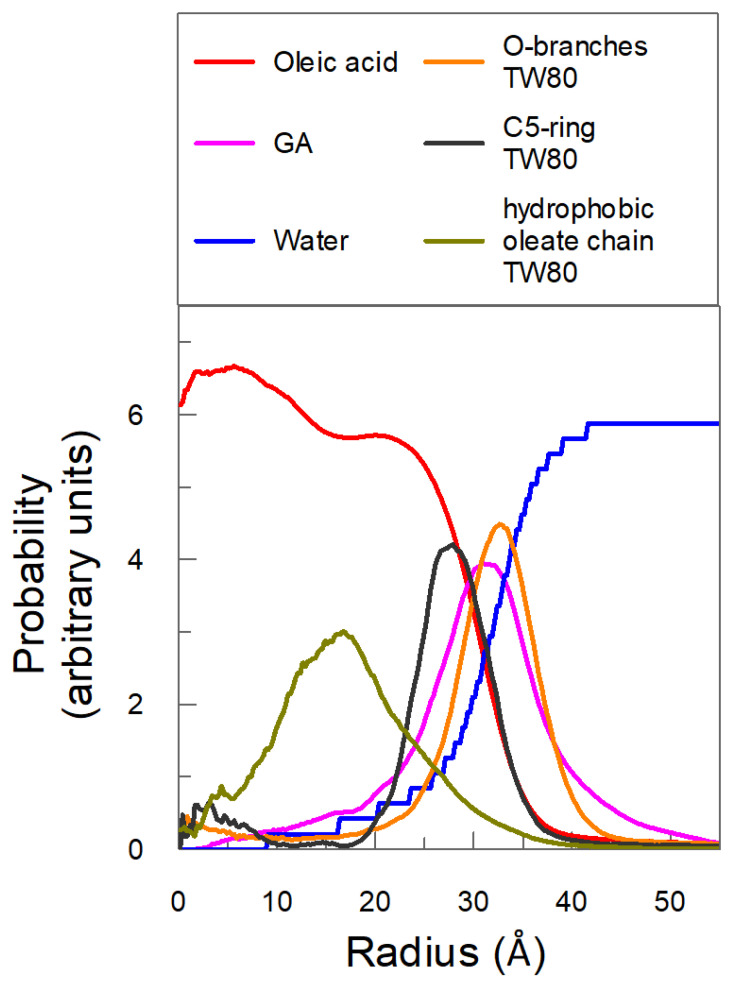
Density of probability of individual components of the nanoemulsion with respect to the center of mass of the oil core of the nanoemulsion droplet (COM).

The location and partitioning of GA were determined in silico by employing molecular dynamics (MD) simulations. An MD production run of 200 ns was computed with eight molecules of GA located randomly in the interfacial region (see Section 3.6) and the spatial configuration of the emulsified system was stored for analysis every 2 ps. Figure 5 shows the variation in the probability of finding oleic acid, Tween 80, water and GA molecules with the distance from the COM of the oil core of the nanoemulsion droplet (R).

Results in Figure 5 show that most of the oleic acid is located with a distance of less than 20 Å, constituting, therefore, the oily core of the nanoemulsion. It is worth noting that the probability of finding oleic acid decreases drastically at distances longer than ~20 Å, and is basically null when R > 35 Å. Figure 5 also shows that the probability of finding water molecules increases sharply at distances > 25 Å and reaches a constant value when R ≈ 42 Å. This sharp variation in the probabilities of oleic acid and water are in keeping with the expected high anisotropy of the system in the boundary between the oil-rich (core) and water-rich regions of the emulsion, and define, therefore, the approximate thickness of the interfacial region that, according to our estimations, is approximately 16–18 Å.

Figure 5 also shows that the probability of finding GA ranges 0–45 Å, with a maximum at 31 Å. This variation is in keeping with the expected high solubility of GA in water, suggesting that a substantial portion of GA is located in the aqueous region of the nanoemulsion. However, the fact that the maximum probability of finding GA is at distances around 30 Å, a distance that is approximately equal to that of the oily core (~20 Å) plus half of the interfacial region (~8.5 Å), suggests that most of the GA is preferentially located in the center of the interfacial region of the nanoemulsion.

MD simulations indicate that the five-carbon ring of Tween 80 is located in the interfacial region close to the oil-rich core, meanwhile the ethylene oxide units of the surfactant are mostly located in the interfacial region pointing towards the aqueous region. The hydrophobic oleate chain of Tween 80 is pointing towards the oil region.

The percentages of GA in the oil, interfacial and aqueous regions were estimated by taking into consideration the radius of the oil core and the thickness of the interfacial region calculated before. The number of gallic acid molecules in each region was determined for each of the 100,000 conformations calculated in the MD simulation and the fraction of GA in each region is expressed as a percentage. Results indicate that most of the GA is located in the interfacial region (~79%), a small fraction (~19%) in the aqueous, and less than 2% in the oil region. The overall picture arising from computer simulations shows that GA is (as expected) basically oil in soluble, and the estimated distribution is in keeping with the experimental values obtained in this work and with reported literature values [11,16,18].

### 4.2. Average Position and Orientation of Gallic Acid Molecules in Oil-in-Water Nanoemulsions

**Figure 6:**
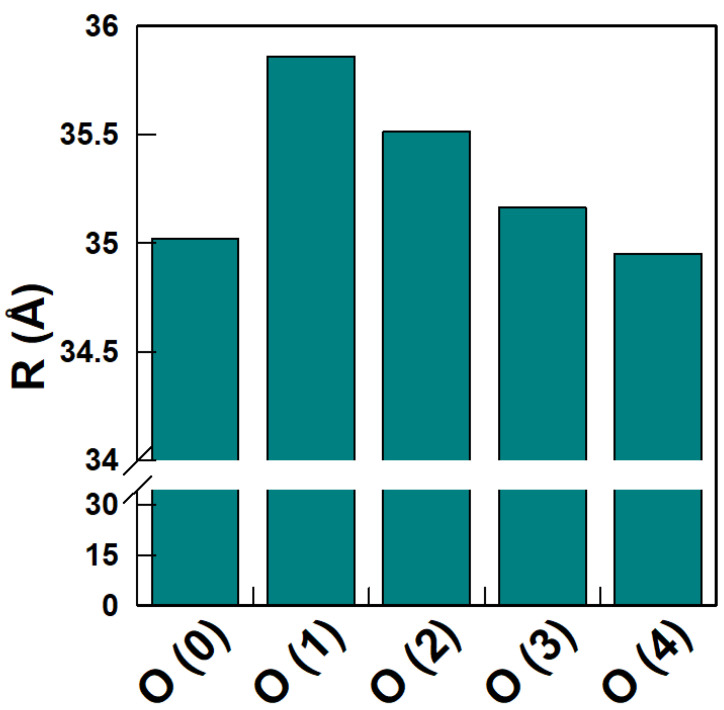
Average distances (Å) between the oxygens of GA molecule and the COM of the nanoemulsion.

The distance (Å) between the oxygen atoms of each GA (Figure 3) and the COM of the nanoemulsion droplet was computed from the stored configurations obtained in the MD simulation. Figure 6 represents the average distance in the simulation for each oxygen.

The results in Figure 6 show that oxygens O(1) and O(2) are further away from the COM of the nanoemulsion than the rest. Results suggest a conformation in which the gallic acid tilts to point with the O(0), O(3) and (O4) oxygens towards the surface of the oil core, while oxygens O(1) and O(2) stay away from it. The obtained distances are in keeping with the results in Figure 5.

**Figure 7:**
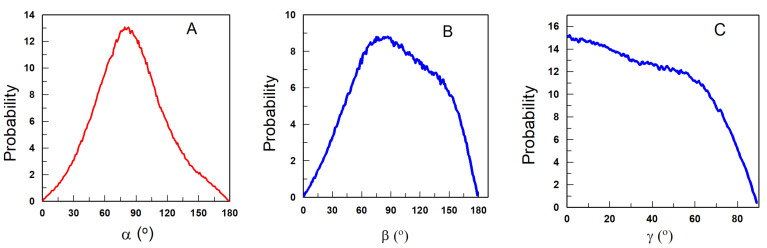
Orientation of gallic acid as defined by angles α (**A**), β (**B**) and γ (**C**).

Figure 7A–C shows the variation in the α (7A), β (7B) and γ (7C) angles that define the relative orientation of gallic acid as obtained from the MD calculations. The values of the angles were obtained for each of the 100,000 configurations stored in the MD calculation and the probability of finding the molecule with a given angle is plotted against the value of the angle, Figure 7. For the sake of clarity, the probabilities are normalized so that the integral under the curve is equal to one (1).

The highest probability for the value of the angle between the COM of the nanoemulsion and the imaginary line containing the O(4)-C4-C1-C7 atoms (α) is given by a well-defined peak at about 80 degrees. The highest probability of the value of angle β is also around 80 degrees, but in this case, the maximum is not so well defined and a range of β angles between 60 and 150 degrees also have high probabilities. Figure 7C shows that the probability for the γ angle decreases slowly from 0 to 60 degrees and more sharply between 60–90 degrees. These values suggest that the molecule adopts an orientation such that the movement of the molecular plane P of the gallic acid (see Figure 3) between 0 and 60 degrees has a very low energy requirement and can be easily adopted under a flip-flop movement. Meanwhile, orientations with γ angles ranging 60–90 degrees are more energetically demanding.

**Figure 8:**
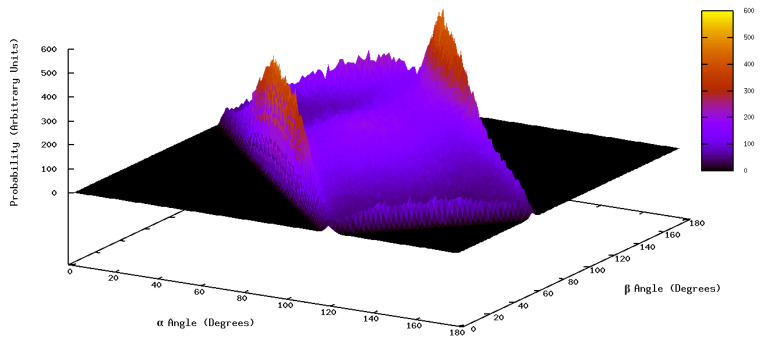
Probability distribution of the combination of α and β angles defined in Figure 3.

When analyzing the combination of the distribution functions of angles α and β, Figure 8, results indicate that there are two separate regions, each one including a maximum of probability. The first one corresponds to α = 55.5 degrees and β = to 64.5 degrees, the second one to α = 82.5 degrees and β = 154.5 degrees. The analysis reflects how each angle changes from its most probable position to find the best combination for both angles, that is, how the values of the angles at the maxima change with respect to those obtained in the distribution of each angle alone.

Considering these results and also those of the obtained distances, we can predict two possible orientations for gallic acid with almost the same probability to appear. The first one is that the O(3) and O(4) atoms are pointing (more or less) to the COM of the nanoemulsion droplet. The second one is that the O(0) atom (instead of the O(3)) is the one pointing to the center of the oil core of the nanoemulsion droplet. Nonetheless, we have to bear in mind that both possible conformations are exchanging continuously; an exchange that is favored by the fact that in both conformations the orientation of the molecular plane with respect to the surface of the nanoemulsion droplet is quite flexible (see Supplementary Materials, Figures S1 and S2).

### 4.3. Oxidative Stability of Nanoemulsions in the Presence and Absence of GA: Reaction Energy Barriers

**Figure 9:**
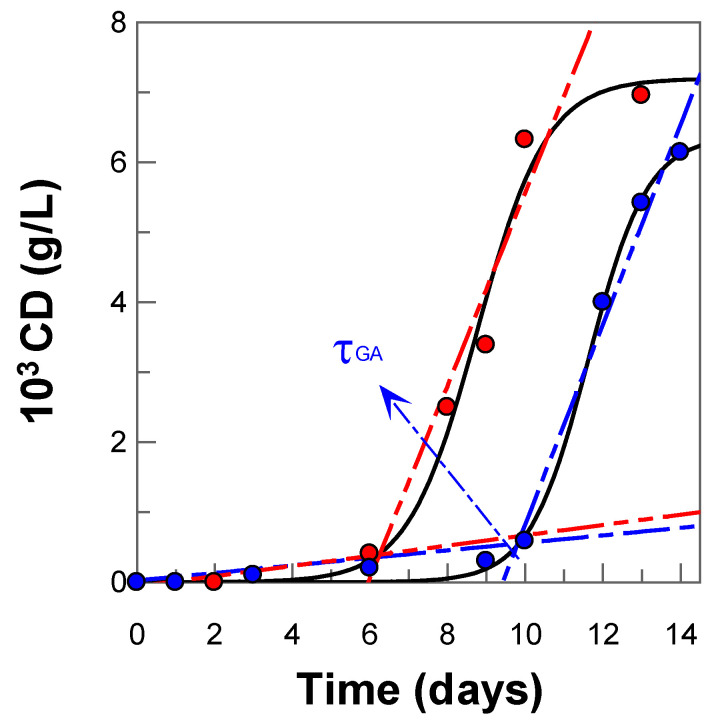
Oxidation kinetic profiles of conjugate dienes (CD) content (g/L) in 1:9 (*v*:*v*) olive-oil-in-water nanoemulsions, T = 60 °C in absence (● control) and presence of gallic acid-(● GA) ([GA_T_] = 10^−4^ M).

The oxidative stability of nanoemulsions was studied experimentally by employing the Schaal oven test, allowing it to spontaneously oxidize in the dark at T = 60 °C. The degree of oxidation was followed, as in previous works, by monitoring the production of primary oxidation products (conjugated dienes, CD, content) with time. The variation in the formation of CDs with time, both in the absence (control) and in the presence of GA, is shown in Figure 9. The kinetic profiles are characterized by an initial relatively slow formation of oxidation products, which corresponds to the initiation step, followed by a rapid formation of CDs (propagation step). As observed, the time necessary to reach the propagation step, defined by the length of the induction period, τ_ind_, is much higher, ~2-fold, in the presence of GA than in its absence, confirming that GA inhibits efficiently the lipid oxidation reaction.

Efficient antioxidants are those whose rate of inhibition (Reaction 5 in Scheme 2) is higher than the rate of propagation (Reaction 3 in Scheme 2) [15,16,45]. According to the theory of absolute rates, the rate of a reaction is closely related to the energy barriers involved in their transition states [26,27], and we deemed it important to investigate the energetics of both the inhibited and non-inhibited reactions theoretically.

The rate of the inhibition reaction, Reaction 5 in Scheme 2, depends on both the effective concentrations of the antioxidant in the interfacial region and on the rate constant of the reaction, *k*_inh_ [15,45]. The effective concentration in the interfacial region was determined by employing Equation (4), bearing in mind the percentage of GA in the interfacial region and the volume of the (interfacial) region, Table 2. The value of the rate constant *k*_inh_ depends on the nature and exact mechanism of the reaction between the antioxidant and the peroxyl radical (Reaction 5 in Scheme 2). Several simultaneous mechanisms are possible, depending on the experimental conditions, but the most frequent is that where an H atom from the antioxidant is transferred to the peroxyl radical ROO^●^ to form the hydroperoxide ROOH and the antioxidant radical.

**Figure 10:**
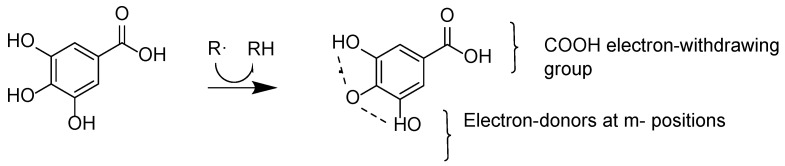
Abstraction of an H atom from the *p* position and stabilization of the resulting radical through intramolecular hydrogen bonding with the *m*-O-H groups [57].

Thus, the rate constant *k*_inh_ depends on the O-H bond dissociation energy, and to obtain insights into the operating mechanisms, we investigated the energetics of the reaction by employing quantum mechanical calculations. Previous B3LYP calculations from our group [56] showed that the O-H bond attached to the C4 (*p* position) is the one which the lowest binding energy value [57] so that once the H atom is transferred, the resulting gallic acid radical is stabilized by intramolecular hydrogen bonding with the two O-H located at the *m* positions (2.152 Å). The resulting GA radical is, thus, characterized by an unpaired electron spin and the charge density is delocalized over the molecule, as illustrated in Figure 10.

**Figure 11:**
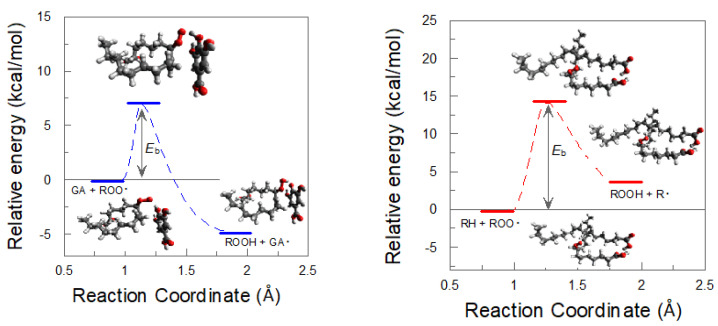
Energy barriers (Eb) profiles and transition states (TS) for the reaction of the peroxyl radical derived from oleic acid, ROO^●^, gallic acid (GA, **left**) and oleic acid (RH, **right**).

We also determined the energy barrier for the reaction between the peroxyl radical ROO^●^ and oleic acid, finding that no significant energy differences were found for the two allylic C-H bonds, resulting in an energy barrier of 14.1 kcal/mol, which is approximately two times higher than that for the H abstraction from GA (7.0 kcal/mol), Figure 11.

Four QM/MM simulations were performed starting from the configurations of the initial, intermediate and final parts of the whole simulation to minimize computation times. In two of these QM/MM simulations, the reaction occurred within the first 20 ps (see Video S1 in Supplementary Section). In principle, the size of the barrier shown in Figure 11 would be a hindrance big enough to prevent the reaction from happening. However, these results are in the gas phase. What the QM/MM results suggest is that the effects that occur in the condensed phase (the interaction with the rest of the molecules, cooperative effects, many body effect, etc.) decrease the size of the barrier, which, combined with thermal effects, makes the reaction possible.

Quantum mechanics results suggest, therefore, that—energetically—the inhibition reaction is favored compared to the propagation reaction. However, the rates of both the inhibition and propagation reactions depend not only on the energy barrier but also on the effective concentrations of the reactants at the reaction site, which is the interfacial region, as shown in Figure 1. Knowledge on the effective concentration of the antioxidant is important because even though the reaction may be favored energetically, the rate of the inhibition reaction (Reaction 5 in Scheme 2) may be lower than that of the propagation (Reaction 3 in Scheme 2) if the effective concentration of the antioxidant is not high enough.

Our results suggest, therefore, that GA is an effective antioxidant because: (1) Its effective concentration in the interfacial region is much higher than the stoichiometric concentration; and (2) The energy required for the reaction with the peroxyl radical to occur is much lower than that between the peroxyl radical and the unsaturated lipid.

## 5. Conclusions

We have employed molecular dynamics simulations and quantum mechanics to mimic an O/W emulsified system and to obtain insights into the position, orientation and distribution of gallic acid and into its reactivity with peroxyl radicals in a modelized nanoemulsion droplet. To validate the model and in silico calculations, we compared the results with those obtained experimentally. The results of these hybrid experiments suggest that the simulated nanoemulsion model developed can predict the experimental result providing a trustable model for mimicking emulsion droplets and the reactivity therein. As far as we know, this is the first time that such an investigation is performed, allowing us to obtain insights on the role of antioxidants in inhibiting the lipid oxidation reaction at the molecular level.

We are aware that in this first stage, we only employed oleic acid to model nanoemulsion because it is the main component of olive oil and for the sake of simplicity. This certainly does not reflect the complexity of real food emulsions, but the promising results obtained encourage us (and hopefully others) to increase the complexity of the models to mimic as much as possible real food emulsions. These studies are in progress and will be part of future reports.

Certainly, understanding the mechanism by which lipid oxidation takes place requires knowing the details of all the chemical reactions involved and their influence on environmental conditions. This requires the knowledge of the distribution, relative location, orientation and physicochemical characteristics of each of the reactants present in the emulsion during the course of the lipid oxidation reaction. In this sense, the development of models that describe how AOs and lipid radicals are distributed between the different regions of the emulsions, their relative orientation and the energetics of the reactions are crucial for understanding and selecting efficient antioxidants to control lipid oxidation reactions.

## Figures and Tables

**Table 1:** Composition (given as percentage in weight) of the main fatty acids of the olive oil employed in this work (EPA: eicosapentaenoic acid, DHA: docosahexaenoic acid).

	Saturated Fatty Acids	Palmitoleic 18:1 ω-7	Oleic 18:1 ω-9	Linolenic 18:3 ω-3	EPA 20:5 ω-3	DHA 22:6 ω-3
Stripped Olive Oil	16	0.9	72.1	3.7	---	---

**Table 2:** Percentages of GA in the interfacial and aqueous regions of O/W nanoemulsion determined in the intact emulsions by employing the kinetic method [10] at the two limiting emulsifier volume fractions (Φ_I_). The values of the induction times (τ_IND_) in the absence and presence of GA are also displayed.

	Control Φ_I_ = 0.005	GA Φ_I_ = 0.005	GA Φ_I_ = 0.04
%GA_I_	---	34.2	81.2
%GA_W_	---	65.8	18.8
10^3^ (GA_I_) (M)	---	6.80	1.95
τ_IND_ (days)	6	10	---

## Data Availability

Not applicable.

## Supplementary Materials

The following supporting information can be downloaded at: https://www.mdpi.com/article/10.3390/antiox12020484/s1. Figure S1: Probability distribution of the combination of α and γ angles. Figure S2: probability distribution of the combination of β and γ angles. Video S1: reaction simulation video (46 GB).
