# Supplementary material for: Molecular Design of Interfaces of Model Food Nanoemulsions: A Combined Experimental and Theoretical Approach
Source: Antioxidants (Basel). 2023 Feb 14;12(2):484. doi: 10.3390/antiox12020484 (PMC9951901; doi:10.3390/antiox12020484)
Supplement: Supplementary file 1 [file antioxidants-12-00484-s001.zip › Suplementary Figures.pdf]

SUPPLEMENTARY INFORMATION

Molecular Design of Interfaces of Model Food Nanoemulsions: A Combined Experimental and Theoretical Approach

Tamara Martínez-Senra [1], Sonia Losada-Barreiro [1,*], Jose M. Hermida-Ramón [1,*], Ana M. Graña [1] and Carlos Bravo-Díaz [1]

Departamento Química-Física, Facultad de Química, Universidade de Vigo, 36310 Vigo, Spain

* Correspondence: sonia@uvigo.es (S.L.-B.); jose_hermida@uvigo.es (J.M.H.-R.)

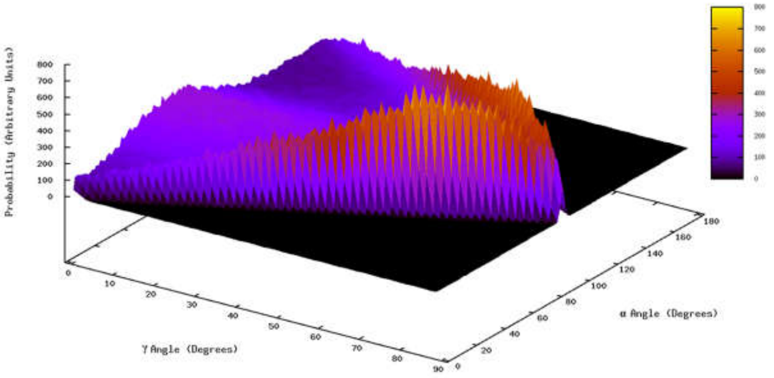

Figure S1. Probability distribution of the combination of α and γ angles.

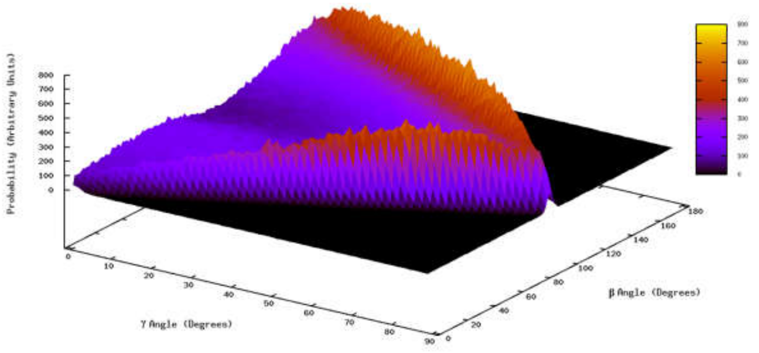

Figure S2. Probability distribution of the combination of β and γ angles.
